# Capacity for delivery of paediatric emergency care and the current use of emergency triage, assessment and treatment in health facilities in the Busoga region, Uganda—A mixed methods study

**DOI:** 10.1371/journal.pgph.0003666

**Published:** 2024-09-04

**Authors:** Goda Laucaityte, Fredrik Wikander Fahnehjelm, Dorothy Akongo, Emmanuel Tenywa, Karl Hildebrand, Moses Kyangwa, Racheal Kwagala Ssemwogerere, William Mugowa Waibi, Helena Hildenwall

**Affiliations:** 1 Department of Global Public Health, Karolinska Institutet, Stockholm, Sweden; 2 Astrid Lindgren Children’s Hospital, Karolinska University Hospital, Stockholm, Sweden; 3 Busoga Health Forum, Jinja, Uganda; 4 Department of Paediatrics, Jinja Regional Referral Hospital, Jinja, Uganda; 5 Department of Clinical Science, Intervention and Technology, Karolinska Institutet, Stockholm, Sweden; 6 Ministry of Health, Kampala, Uganda; 7 SWEDESD- Department of Women’s and Children’s Health, Uppsala University, Uppsala, Sweden; PLOS: Public Library of Science, UNITED STATES OF AMERICA

## Abstract

The implementation of structured guidelines, such as the World Health Organisation’s Emergency Triage, Assessment and Treatment has been shown to reduce in-hospital mortality, addressing the high burden of early in-hospital deaths. We evaluated the capacity to provide paediatric emergency care at higher-level health facilities in the Busoga sub-region, Uganda, and explored healthcare workers’ perceptions of quality care. This assessment aimed to inform policy and facilitate the implementation of guidelines. A comprehensive mixed-methods study was conducted, comprising a facility audit, a survey of healthcare providers to assess their knowledge, and focus group discussions with facility staff. The study included all public and private not-for-profit facilities that provide in-patient paediatric care in Busoga. Quantitative data were analysed using descriptive statistics and linear regression, while thematic analysis with the framework method approach was applied to qualitative data. A total of 14 focus group discussions, 14 facility audits, and 100 surveys with healthcare providers were conducted. Essential equipment for paediatric emergencies and staff shortages were identified as primary barriers to quality care and key contributors to worker demotivation. Referrals were one of the main challenges, with only 25% of facilities accessing a fuelled ambulance. Knowledge scores were higher among healthcare professionals who had undergone emergency management training and participated in refresher courses (mean 13.2, 95% CI 11.6–14.8, compared to 9.2, 95% CI 8.0–10.3). Participants who felt well-prepared achieved markedly higher scores on knowledge surveys than those feeling unprepared (mean 12.2, 95% CI 11.2–13.1, versus mean 8.5, 95% CI 7.3–9.7). Qualitative discussions demonstrated positive attitudes of healthcare workers toward ETAT guidelines. Results underscore the importance of focused training with refresher sessions to enhance health workers’ knowledge and confidence in managing paediatric emergency cases. However, substantial limitations in staffing numbers and the availability of necessary equipment need to be addressed for overall quality of care improvement.

## Introduction

Five million children under five years of age died in 2021, with the majority of deaths occurring in low- and middle-income countries (LMICs) [1]. Leading causes of death outside the neonatal period include preventable and treatable infectious diseases such as lower respiratory tract infections, diarrhoea, and malaria [2]. Child mortality in LMICs continues to be unacceptably high, with a significant proportion of deaths occurring within the first 24 hours of hospital admission [3–6]. Key factors contributing to early in-hospital mortality include delays in seeking care, and the delayed recognition and management of critically ill children [3–6]. Importantly, many of these deaths are preventable, and weaknesses in healthcare systems, particularly in unstructured triage systems and limitations in the delivery of quality emergency care, have been identified as contributing factors in low-resource settings [7–9].

Well-organised acute paediatric care, including early resuscitation and stabilization, can significantly reduce childhood mortality [7, 9, 10]. This can be achieved by the implementation of evidence-based guidelines and the provision of training to health workers along with reliable supplies of the required equipment [10–12]. The World Health Organisation (WHO) developed the Emergency Triage, Assessment and Treatment (ETAT) clinical guidelines as a guidance to improve the early recognition and management of children presenting with life-threatening conditions in resource-limited settings [13, 14]. Individual countries in East Africa have then adapted the guidelines according to the local context and expanded them further into ETAT+, which contains additional material on newborn resuscitation, and supportive care during admission and also covers some other common causes and outcomes of severe diseases in the neonate or child [15]. Kenya, Rwanda, Madagascar, Malawi, and Sierra Leone, among others, have already implemented the aforementioned triage and treatment algorithms in some of their hospitals, resulting in reduced in-hospital child mortality [15–19]. However, the capacity of hospitals, current knowledge, and practice in Uganda to deliver paediatric emergency care have not yet been assessed. Thus, the barriers and facilitators to implementing ETAT+ in Ugandan hospitals are not well known. This study was developed to explore healthcare workers’ (HCWs) experiences in managing paediatric emergency cases, assess hospitals’ capabilities, and identify the primary challenges in implementing the guidelines.

## Materials and methods

We carried out a mixed-methods study with a concurrent triangulation design, collecting data simultaneously [20]. Quantitative data included facility inventories to evaluate the availability of essential equipment and drugs for paediatric emergencies (S1 File) and quantitative surveys assessing the knowledge of HCWs managing paediatric emergencies (S2 File). Qualitative data were obtained via Focus Group Discussions (FGDs), exploring existing practices and HCWs’ perspectives on ETAT+ guidelines’ implementation (S3 File). As Uganda did not have its own ETAT+ guidelines at the time of data collection, information on existing practises and training focused on the original ETAT, while the readiness component considered the implementation of ETAT+ in triage and emergency room management procedures. Thus, the FGDs’ predefined topic guide consisted of four different topics: current emergency treatment, referral procedures, ETAT awareness, and ETAT+ preparedness.

### Setting

Data were collected in the Busoga sub-region, located in the central eastern part of Uganda. According to the latest report published in 2021 by the Uganda Bureau of Statistics, the Busoga sub-region was ranked as the poorest in the country. An estimated 4 million people live in Busoga, with a significant proportion being children and adolescents. The under-five mortality rate in the sub-region is higher compared to the country’s average, reaching 84 deaths per 1,000 live births [21]. Data were collected from all public and private not-for-profit (PNFP) health facilities providing inpatient paediatric care in Busoga, including one regional referral hospital, four general hospitals, two PNFP hospitals, and seven level IV healthcare centres (14 total).

### Data collection and selection of participants

The study data consisted of 14 facility inventories, 100 individual surveys with health workers, and 14 FGDs with healthcare staff of different cadres providing emergency paediatric care at various facilities within the sub-region. The inventory questions were derived from selected sections of the WHO’s Hospital Care for Children: Quality Assessment and Improvement Tool, focusing on required items to adhere to ETAT+ guidelines [22]. They covered availability of infrastructure, drug stocks, equipment availability, staffing and referral capacity. The survey was based on questions used in ETAT+ training materials and covered participants’ experience, training, knowledge of ETAT/ETAT+, and current clinical practice, including referrals. The survey included 14 knowledge questions, with 0–2 points assigned to each question.

The inventories, FGDs, and initial surveys were conducted over one week from the 31^st^ of January to the 5^th^ of February, 2023, with additional surveys completed in April 2023. A team of data collectors visited one facility at a time, completing the inventory, surveys, and FGDs before moving on to the next facility. The facility in charge was chosen to respond to the inventory questions. The surveys were conducted with HCWs who regularly manage children, capturing individual experiences and knowledge in managing severely sick children. Quantitative data were collected using RedCap on handheld mobile devices, with data uploaded to the server at the end of each workday [23, 24].

The FGD tool was developed to cover aspects of emergency care that are not easily quantified, such as feelings of support, motivation, and confidence. The topic guide was developed using results from previous work on health workers’ experiences and perceptions of emergency care delivery [25, 26]. FGDs lasted approximately 30–45 minutes. For logistical reasons, i.e. the shortage of staff and time constraints, one discussion per facility was conducted with four to eight HCWs of different cadres per discussion. For participant recruitment, purposeful sampling was used, including healthcare providers who regularly care for children to yield the most relevant information. The approach was also pragmatic, selecting all staff members managing severely sick children available for discussion during the study period. The FGDs were facilitated by a moderator, a Ugandan social scientist with prior experience in qualitative data collection. In addition to the moderator, a note taker captured the verbal and non-verbal interactions of participants in the discussions. The language used was either English or the local language Lusoga, as preferred by the participants. The discussions were recorded, transcribed by the social scientist fluent in both Lusoga and English, translated into English when needed, and anonymised before sharing with the rest of the study team.

### Analysis

Quantitative data were transferred to STATA v.16 (StataCorp LLC, College Station, TX) for analysis, then summarized using proportion percentages, and medians [27]. The data was categorized based on facility category and healthcare provider classification, as applicable. The responses from 14 survey questions related to knowledge were combined into an overall knowledge score, with a maximum total score of 28 points. This score was used for a linear regression analysis to assess links between HCWs’ knowledge scores and their training history, years of experience and professional category. P-values less than 0.05 were considered statistically significant.

The FGDs data were analysed using NVivo version 14.0 (Release 1.7.1) software, employing thematic analysis with a pragmatic framework approach [28, 29]. This systematic yet flexible method enables the identification of patterns in the data and generation of the framework matrix [29]. The content was analysed inductively, with codes and categories formed during the coding process. Analysis involved following these consecutive steps: 1) transcription, 2) familiarisation, 3) coding, 4) developing an analytical framework, 5) applying the analytical framework, 6) charting data into the framework matrix, and 7) interpreting the data [29]. Initial coding of five FGDs was done by GL. The codebook was then shared and discussed with HH and DA, codes were updated accordingly, and applied. Themes were then developed and discussed with a wider study team, several of whom are working within the study facilities, to agree on the final interpretations.

### Ethical considerations

This study has been reviewed by the Higher Degrees Research and Ethics Committee of Uganda Christian University, Mukono (UCUREC-2022-397). All the participants were provided with comprehensive information about the study prior to obtaining the written consent from each. Participants were given the option to skip questions if they did not want to address them or withdraw from the discussion entirely if they felt uncomfortable at any time. All the data were kept confidential, and only anonymised versions were shared with other members of the research team. Moreover, the audio recordings and the transcriptions were encrypted and held on study laptops protected with a password, whereas all paper consent forms were stored in a locked room accessible only to members of the study team.

## Results

The healthcare providers in this study included paediatricians, medical officers, clinical officers, nurses, midwives, and laboratory personnel managing children with emergency needs (Table 1).

**Table 1:** Description of the sample of participants including their profession.

**Quantitative survey**								
Cadre/ Facility	Paediatrician	Medical Officer	Clinical Officer	Nurse	Nurse Assistant	Midwife	Other	Total
PNFP^a^	0	0	6	3	0	6	0	15
Public	2	12	15	26	3	25	2	85
Total	2	12	21	29	3	31	2	100
**Qualitative FGDs** ^**b**^								
Cadre/ Facility	Intern Doctor	Medical Officer	Clinical Officer	Nurse	Nurse Assistant	Midwife	Other	Total
PNFP^a^	0	2	0	2	0	9	0	13
Public	1	7	11	32	2	16	8	77
Total	1	9	11	34	2	25	8	90

^a^PNFP–private not-for-profit

^b^FGDs–Focus Group Discussions

**Fig 1:**
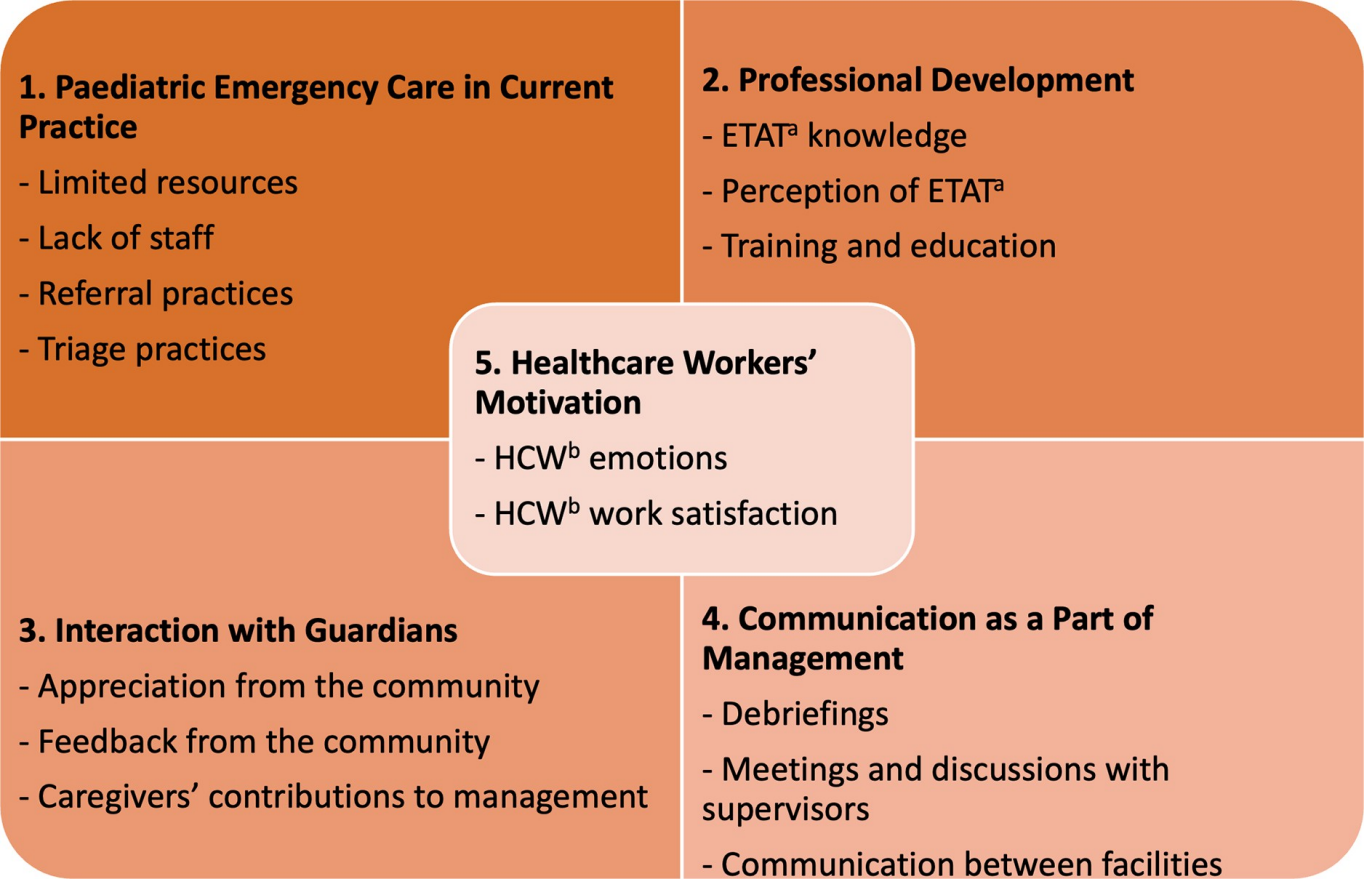
Themes and subthemes. ^a^ETAT–Emergency Triage Assessment and Treatment ^b^HCW–Healthcare Worker.

Five themes were identified as the main areas of importance for readiness to deliver paediatric emergency care: 1. Paediatric Emergency Care in Current Practice 2. Communication as a Part of Management 3. Interaction with Guardians 4. Professional Development, and 5. Healthcare Workers’ Motivation. The qualitative and quantitative results are presented together under each of the five themes with subsequent subthemes (Fig 1). Healthcare Workers’ Motivation is placed centrally in the framework as it is highly influenced by other themes.

### 1. Paediatric emergency care in current practice

All facilities reported shortages of supplies needed for emergency care, with only 33% of public facilities reporting the availability of IV Artesunate and Ceftriaxone at the time of data collection. Table 2 provides an overview of the available equipment and drugs by facility ownership.

**Table 2:** Equipment and drug availability across different types of institutions.

Material	PNFP^a^	Public	Total
	N = 2 n (%)	N = 12 n (%)	N = 14 n (%)
Emergency trolley available	2 (100)	7 (58)	9 (64)
^•^ Content defined	2 (100)	4 (33)	6 (43)
^•^ Required items available	2 (100)	2 (17)	4 (29)
Functioning glucose monitor	2 (100)	4 (33)	6 (43)
Pulse oximeter, any	2 (100)	11 (92)	13 (93)
^•^ Adult probe	2 (100)	7 (58)	9 (64)
^•^ Paediatric probe	1 (50)	10 (83)	11 (79)
Oxygen available	2 (100)	12 (100)	14 (100)
Resuscitation bag size 1–2	2 (100)	9 (75)	11 (79)
Resuscitation bag size 3–4	2 (100)	5 (42)	7 (50)
NG-tube adult size (10–12)	1 (50)	7 (58)	8 (57)
NG-tube, any paediatric size (6–8)	2 (100)	6 (50)	8 (57)
Hb testing	2 (100)	10 (83)	12 (86)
Cross matching	2 (100)	12 (100)	14 (100)
Infusion set	2 (100)	7 (58)	9 (64)
Chest drain	0 (0)	3 (25)	3 (21)
Guedel ped	1 (50)	10 (83)	11 (79)
Guedel neo	1 (50)	7 (58)	8 (57)
Suction pump	2 (100)	9 (75)	11 (79)
Nebulizer	2 (100)	5 (42)	7 (50)
**Emergency drugs**			
Adrenaline	2 (100)	8 (67)	10 (71)
Diazepam	2 (100)	11 (92)	13 (93)
Phenobarbitone	2 (100)	8 (67)	10 (71)
Phenytoin	1 (50)	6 (50)	7 (50)
IV-fluids other than D10/25/50	2 (100)	6 (50)	8 (57)
^•^ NaCl	2 (100)	5 (42)	7 (50)
^•^ Ringer’s lactate	1 (50)	5 (42)	6 (43)
^•^ Half Darrow 5% glucose	1 (50)	4 (33)	5 (36)
^•^ D10 or D50	2 (100)	8 (67)	10 (71)
**Antibiotics**			
^•^ Benzyl PC	2 (100)	6 (50)	8 (57)
^•^ Gentamicin	2 (100)	5 (42)	7 (50)
^•^ Ceftriaxone	2 (100)	4 (33)	6 (43)
^•^ Ampicillin	2 (100)	6 (50)	8 (57)
^•^ Cloxacillin	2 (100)	2 (17)	4 (29)
IV Artesunate	2 (100)	4 (33)	6 (43)
Dexamethasone/Hydrocortisone	2 (100)	7 (58)	9 (64)
Salbutamol nebulizer	2 (100)	2 (17)	4 (29)
Ipratropium	1 (50)	0 (0)	1 (7)
Aminophylline	2 (100)	6 (50)	8 (57)
Magnesium Sulphate	2 (100)	9 (75)	11 (79)
F75/100	1 (50)	3 (25)	8 (57)
ORS^b^	2 (100)	6 (50)	8 (57)
ReSoMal	1 (50)	0 (0)	1 (7)

^a^PNFP–private not-for-profit

^b^ORS–Oral Rehydration Solution

The qualitative discussions revealed that in addition to the lack of equipment, dysfunctional items were sometimes provided, and these challenges were reported across all facility levels.

• *“Our pulse oximeter sometimes it is on and off, sometimes it gives good results other times wrong results because it is faulty.”—General Hospital.*

Four of the facilities (29%) were staffed with at least one paediatrician. Due to insufficient staffing HCWs were forced to work beyond their assigned roles.

• *“Limitations of health workers is the biggest issue in Uganda. You find that you are the midwife, you are the counsellor, doctor, health educator but the beauty is if you are doing things to save life, everyone joins in, even the cleaners are part of the emergencies.*”—*General Hospital.*

Referrals posed a major challenge to successful management. Children were referred due to facility limitations like a lack of specialized doctors (specifically, a paediatrician), supply shortages, or the need for higher-level care. Incomplete referrals were mainly due to transportation issues or fuel shortages. Notably, only 25% of public facilities had access to a fuelled ambulance.

• *“They didn’t have transport to Jinja, and, in the ambulance, there was no fuel. We were stuck there; we were looking at each other with nothing to do and the child was in a critical condition.*”—*General Hospital.*

All but two staff (98%) reported that their facility used some sort of triage, most commonly the general appearance of the child (35%) but Integrated Management of Childhood Illness (IMCI) (32%) and ETAT (24%) were also reportedly used. Among benefits of triaging, the ability to deal with patients with critical conditions quicker and appreciation from the community were mentioned.

• *“I appreciate because at times there are emergencies like in [Outpatient Department] they have gone as far as getting different [colour coded] stamp, like for a seriously ill child, they use a red stamp, and you are able to identify that this patient needs serious attention*.”—*General Hospital.*

67% of HCWs reported managing an emergency case within the last week. 76% managed with 2–3 people, and 31% had all necessary items available.

### 2. Communication as a part of management

All respondents reported that they had someone to consult when handling paediatric emergency cases. Of these, 53% responded that consultations were always possible, 19% reported "most of the time," and 28% said "sometimes." As a benefit of discussing with superiors, the HCWs mentioned the ability to improve and make positive changes in clinical practices.

In the qualitative interviews, receiving feedback from supervisors was mostly perceived as the supervisor’s ability to respond to HCWs requests in terms of tools, equipment, or additional staffing.

• *“So, when we need to be supplied with power, we call the administration and when they respond it is positive feedback from the administration*.”— *General Hospital*.

According to the participants, flaws of communication between facilities and scarcity of feedback resulted in compromised management, not being updated about their patients’ conditions, and a feeling of stagnation in professional development.

• *Then too we have a challenge with feedback, we don’t get feedback from the referred points, we refer but getting feedback is still <…>, I don’t know whether my patient improved or not*.”— *Health Centre IV.*

A third (32%) of respondents reported having debriefings after managing emergency cases. This was reportedly done during department meetings, continuous medical education (CME) meetings, quality improvement committee meetings, or audits.

• *“We tend to have meetings with our departmental in-charges where we are able to raise our concerns that we are facing. When they can, we get the solutions and when they can’t, that is the situation, we are in*.”—*Health Centre IV.*

### 3. Interaction with guardians

A total of 66% of respondents reported receiving regular appreciation. The primary sources of appreciation were patients (54%) and the general community (25%), with additional acknowledgment from colleagues (38%) and superiors (41%). In some facilities, village health teams worked as communicators between HCWs and the community, holding regular meetings and reporting feedback.

• *“Yes, we get, there are those ones who appreciate, they come back and tell us that the services have improved compared to those days.*”—*Health Centre IV.*

HCWs were forced to ask caregivers to contribute financially, thus the ability to provide care was oftentimes dependent on the financial situation of the caregiver, which in turn had an impact on the outcome.

• *”We want to help the patient, we have to talk to care takers and see if they can buy so that we are able to so that we are able manage the child and if they refuse still, we have nothing much to do.*”—*Health Centre IV.*

### 4. Professional development

Of the participants, 47% had undergone any paediatric emergency care focused training, including ETAT, Advanced Paediatric Life Support (APLS), or Paediatric Advanced Life Support (PALS). Additionally, 29% had received refresher training, and 42% had engaged in simulation training.

Familiarity with the ETAT guidelines varied both across different facilities and within individual facilities. Participants who were familiar with the ETAT guidelines cited benefits such as reduced referrals, minimised delays, improved healthcare skills, and saved lives. They identified obstacles like shortages of critical supplies and suggested solutions such as ensuring availability of equipment and drugs, along with continuous training and supervision.

• *“We would advocate more for refresher training, supplies and space because some things are out of our hands and also motivation for the people who are implementing.*”—*Regional Referral Hospital.*

Notably, 51% health workers reported never receiving any form of training in paediatric emergency care. Another issue identified during qualitative interviews was the frequent turnover or relocation of trained staff.

Among all included HCWs, the median knowledge score was 10 (IQR 2–18). Knowledge scores were primarily increased with received training and refresher trainings (Table 3). Healthcare professionals who had undergone emergency management training and participated in refresher courses had a mean score of 13.2 (95% CI 11.6–14.8), compared to 9.2 (95% CI 8.0–10.3) among HCWs without emergency care training. The full results of regression analysis can be found in the supplementary file (S4 File).

**Table 3:** Linear regression analysis of ETAT knowledge.

Variables		Unadjusted Analysis			Adjusted Analysis		
		coefficient	95% CI^a^	p-value	coefficient	95%CI^a^	p-value
Profession	Paediatrician	Ref			Ref		
	Medical Officer	-2.8	-8.0–2.5	0.300	-0.9	-5.9–4.2	0.738
	Clinical Officer	-4.3	-9.4–0.7	0.091	-2.0	-7.0–3.0	0.432
	Nurse	-6.3	-11.2- -1.2	0.015	-4.3	-9.2–0.6	0.087
	Nurse Assistant	-14.5	-20.8–8.2	<0.001	-11.6	-17.8- -5.3	<0.001
	Midwife	-8.1	-13.1- -3.1	0.002	5.7	-10.1- -0.8	0.024
	Other Training	-3.4	-12.9–0.9	0.086	-3.3	-10.0–3.4	0.333
Years duty	<1 year	Ref			Ref		
	1–3 year	0.4	-2.2–3.0	0.738	-0.3	-2.5–2.0	0.805
	>3 years	2.7	0.9–5.1	0.036	1.3	-1.0–3.5	0.252
Emergency Training	None	Ref			Ref		
	ETAT^b^/APLS^c^/ PALS^d^/Other	1.2	-0.9–3.3)	0.280	0.7	-1.0–2.5	0.421
	ETAT^b^/APLS^c^/ PALS^d^/Other + refresher training	3.5	1.7–5.3	<0.001	2.3	0.7–4.0	0.007

^a^95%CI– 95% confidence interval.

^b^ETAT–Emergency Triage Assessment and Treatment.

^c^APLS–Advanced Paediatric Life Support.

^d^PALS–Pediatric Advanced Life Support.

### 5. Healthcare workers’ motivation

Overall, the interviews revealed that having what was needed to deal with a paediatric emergency case led to better care, fewer referrals and HCWs’ contentment and self-satisfaction. On the contrary, inability to provide care due to limitations of equipment and medicines resulted in frustration and despair among health workers. HCWs expressed their effort to do whatever was possible despite the limitations using phrases such as “did my best”, “we tried our level best”, “it is not our fault”.

• *“Ok I did my best but only that some of the things were not around like cannulas, syringes and some antibiotics.*”—*Health Centre IV.*

The median self-reported work satisfaction among HCWs, was 6 (IQR 4–9) on a scale of 0–10. 51% of participants reported feeling prepared or well-prepared to manage paediatric emergency cases, and this feeling was associated with a higher self-scoring of work satisfaction (mean 6.9, 95% CI 6.5–7.2 versus mean 6.0, 95% CI 5.7–6.4). To add more, participants who felt well-prepared (51%) scored higher on the knowledge survey (mean 12.2 (95% CI 11.2–13.1) than those responding negatively (49%) (mean 8.5 (95% CI 7.3–9.7).

• *“Yes, I was telling you that the benefits are on both sides of the patient and the health worker. When the patient gets the services, it will save life and also if I see my patient getting better, I feel good*.”—*Health Centre IV.*

While not statistically significant, health workers who reported they felt appreciated had a median knowledge score of 11 (IQR 4–18), whereas those who did not feel appreciated had a median knowledge score of 10 (IQR 3–15). Additionally, no discernible differences were observed in work satisfaction based on whether respondents felt appreciated or not, or from whom the appreciation originated.

## Discussion

This study aimed to assess the current capacity for providing paediatric emergency care and provides a baseline understanding for facility readiness to implement ETAT+ in health facilities in Busoga, Uganda. The main findings reveal significant challenges in the availability of drugs and equipment necessary for managing severely sick children, thereby limiting the ability of health workers to deliver quality care. Despite these institutional shortcomings serving as demotivating factors, there is an observable increase in work satisfaction when participants feel more prepared. The emotions and motivation of HCWs are positively influenced by opportunities for communication with peers, superiors, and community members. Moreover, health workers who had undergone both training and refresher courses performed better on knowledge assessment compared to those with less training. Higher knowledge scores were also observed among staff who self-reported feeling prepared to manage critically ill children.

### Organisational factors

Institutional limitations, such as shortage of medicines, equipment, and medical supply, along with understaffing, hinder the provision of quality care and jeopardize the successful implementation of ETAT+. These constraints are prevalent in low-resource settings and have been identified as demotivating factors in other studies [30–32]. Our findings also support previous reports of equipment and drug shortages [25, 26, 33]. A survey by Ugandan authorities highlighted significant lack of material and drugs, particularly in eastern Uganda [34]. Working without basic equipment not only demotivates HCWs but also contributes to HCW turnover [35] and deters young medical professionals from working in rural areas [36]. These challenges impact HCWs’ morale [37], leading to dissatisfaction [38] and increased workload [39].

Aside from the material shortages, insufficient human resources were also recognized as a challenge, leading to heavy workloads and negatively impacting worker motivation [40]. Among the 14 facilities in our study, only four had a paediatrician on their team. The lack of specialized medical workers is a global challenge, with LMICs bearing the greatest burden of understaffing [41].

Referring a child to another facility, when necessary, proved challenging, with only a quarter of the public facilities having access to a fuelled ambulance. Previous studies have demonstrated the high cost of referrals and transportation challenges as some of the weakest parts of emergency care in low-resource settings [26, 42]. Unreliable communication between facilities for organising referrals and underdeveloped referral systems were additional challenges. A qualitative study in rural Uganda revealed that guardians perceive ambulances as unreliable due to poor communication between the vehicle driver, healthcare centre and caregivers [42].

Efficient communication between colleagues and supervisors was not always reported. Lack of communication is a known precursor to medical errors [43]. Supervision and audits with constructive feedback can enhance worker performance, whereas poor communication between HCWs and managers negatively impacts worker motivation [44, 45]. We found no difference in workers’ satisfaction levels based on whether appreciation was felt even though it is consistently described in previous studies [46–49]. Improving teamwork, fostering debriefing practices, and conducting regular meetings can enhance worker performance without significant expense.

As feedback loops were ineffective, participants in our study sought alternative ways to stay updated on referred patients’ conditions. The desire for feedback resonates with findings from previous research where HCWs saw it as necessary for their professional development, even if 79% of them did not actively follow up with patients [26]. Comparably, a study in Tanzania revealed frustration when subsequent feedback after a referral was lacking, resulting in missed learning opportunities [50]. Developing referral networks and creating effective feedback loops are essential reforms for facilitating management and promoting learning opportunities.

### Community and clients

The community context is critical to health workers’ motivational processes through its impact on worker experience of outcomes [51]. Strengthened connections with communities lead to increased feedback, enhancing worker performance [47]. Being appreciated by the community serves as a significant motivator, as found in studies in Tanzania and western Nigeria [52, 53]. A systematic review identified twelve studies confirming that good community relations increase motivation, underscoring the importance of social factors [30]. Caregivers are not only expected to but also directly contribute to the management of critically ill children, particularly in referrals. Lack of funds and transportation difficulties were significant barriers for caregivers to complete referrals in rural Uganda [42]. As emphasised in a study conducted in Kampala, high out-of-pocket expenditures serve as another barrier to accessing emergency care services [5].

### Organisational efforts to improve workers capability

The evidence illustrates that HCWs value training opportunities and knowledge acquisition, consistent with prior studies [30, 31, 50, 54]. A comprehensive systematic review on HCWs motivation in the East African community highlights the importance of continuous education and training throughout one’s career [32]. In our study, health workers acknowledged the significance of training refreshment in enhancing performance on ETAT knowledge. However, HCWs also expressed frustration over possessing knowledge but being unable to utilize it due to material shortages required to adhere to guidelines [25, 55]. Inadequate organisation can exacerbate staff shortages and create workload imbalances, generating excessive workloads [37]. Disparities in training accessibility exist across healthcare cadres and sectors with workers from private sectors having fewer opportunities [37, 39, 45, 52]. Ensuring dissemination of knowledge is crucial for the successful adoption of new guidelines.

## Worker experience of outcomes

In this study, HCWs expressed negative feelings about the lack of supplies and equipment for patient management, posing challenges to care provision. On the contrary, HCWs were satisfied and content when able to provide quality care successfully. Feelings of despair and burnout due to high workloads and lack of medical equipment increase the risk of poor performance [45]. Remarkably, participants who felt prepared not only scored higher on the knowledge survey but also reported greater job satisfaction. Kitsios and Kamarioutou’s study in Greece identified work achievements among key motivators for public health workers’ satisfaction [56]. Job satisfaction predicts worker motivation and system stability, while dissatisfaction is associated with HCW turnover [49, 57]. We found that a sense of preparedness to manage paediatric emergencies predicts higher job satisfaction scores, likely due to consistent staff training, a steady supply of resources, and the ability to consult with colleagues.

## Methodological limitations

This study has limitations that may impact the interpretation of findings. Firstly, facility audits were conducted on a single day, providing only a snapshot of drug and equipment availability. Secondly, focus groups were organised by facility, resulting in mixed cadres in each group. This mix of participants holding different positions could introduce power dynamics, potentially leading some participants to withhold their opinions. However, the moderator actively encouraged all participants to engage, mitigating potential bias. Additionally, health district managers did not participate, reducing the potential stress for participants to freely share their experiences. Not involving district health managers or patients is a limitation, as their input could have enriched the findings.

## Conclusions

To the best of our knowledge, this is the first study exploring barriers to implementing ETAT+ as perceived by HCWs in Uganda. It may provide valuable insights for the ongoing plans to implement ETAT+ nationally in Uganda. While not all participants were familiar with ETAT or ETAT+, it was perceived as providing numerous benefits if fully adopted. The results indicate that regular training significantly improves workers’ knowledge and readiness to manage paediatric cases, leading to increased job satisfaction. Establishing referral networks and implementing effective feedback loops are desired reforms for enhancing management and providing learning opportunities. Implementing other strategies, such as enhancing teamwork, fostering debriefing practices, and holding regular meetings can boost worker performance at a low cost. Additionally, building closer bonds with the community and creating opportunities to receive feedback from caretakers also serve as motivational factors for providing quality care.

While several opportunities for implementation were identified, our findings also highlight the insufficient supply of medical equipment and medicines as a major obstacle to providing quality paediatric emergency care. ETAT+ cannot be successfully implemented unless the required resources are available to HCWs when needed, underscoring a broader systemic issue that demands attention.

## Supporting information

S1 Checklist (DOCX)

S1 File Facility audit. (PDF)

S2 File Health worker questionnaire. (PDF)

S3 File Topic guide. (PDF)

S4 File Regression analysis. (DOCX)
