# Supplementary material for: Capacity for delivery of paediatric emergency care and the current use of emergency triage, assessment and treatment in health facilities in the Busoga region, Uganda—A mixed methods study
Source: PLOS Glob Public Health. 2024 Sep 4;4(9):e0003666. doi: 10.1371/journal.pgph.0003666 (PMC11373804; doi:10.1371/journal.pgph.0003666)
Supplement: S1 File — (PDF) [file pgph.0003666.s002.pdf]

Facility audit

Record ID ______________________

Data Collector ______________________

Hospital name ______________________

Emergency room or ward?
- ☐ Emergency room
- ☐ Ward

Today's date ______________________

General Facility Resources

Facility ownership
- ◯ Public
- ◯ NGO
- ◯ Private
- ◯ Private not for profit

Power supply
- ☐ Grid
- ☐ Generator
- ☐ Back-up batteries
- ☐ Solar panels
- ☐ Other
- ☐ None

Inpatient facilities
- ☐ Paediatric ward
- ☐ Maternity unit
- ☐ Neonatology unit
- ☐ General ward
- ☐ Side room
- ☐ None

Opening hours
- ◯ Staffed 24/7
- ◯ Staffed during day + on call
- ◯ Staffed in morning + on call
- ◯ No on call

Is there staff-accommodation on-site?
- ◯ Yes
- ◯ No

How long time does it take for the on-call to reach the facility (minutes)? ______________________

Has the hospital experienced any power cuts
- ◯ Today
- ◯ Last 24 hrs
- ◯ This week
- ◯ This month
- ◯ >one month ago

What is the hospitals main source of blood?

- ◯ Hospital blood bank
- ◯ Central blood bank
- ◯ No blood provided at facility

Emergency Euipment

ask for each if available and functioning

Is there an emergency trolley or similar?

- ◯ Yes in emergency room
- ◯ Yes in ward
- ◯ Yes in both emergency and in ward
- ◯ No

Is content of emergency trolley clearly defined somewhere (please show document)?

- ◯ Yes
- ◯ No

Are the required items available in the emergency trolley today?

- ◯ Yes
- ◯ No

Blood glucose monitor

- ◯ Available and functioning
- ◯ Available but not functioning
- ◯ Not available

Glucose test strips

- ◯ Available and functioning
- ◯ Available but not functioning
- ◯ Not available

Pulse oximeter with probe

- ◯ Available and functioning with adult probe
- ◯ Available and functioning with paediatric probe
- ◯ Available and functioning with paediatric and adult probe
- ◯ Available but not functioning
- ◯ Not available

Oxygen

- ◯ Yes in ER only
- ◯ Yes in ward only
- ◯ Yes both in ER and ward
- ◯ No

How many patients can be put on oxygen at the same time?

____

Nasal prongs

- ◯ Available adult size
- ◯ Available paediatric size
- ◯ Available neonatal size
- ◯ Available all sizes
- ◯ Not available

Resuscitation bag+mask size 1

- ◯ Available and functioning
- ◯ Available but not functioning
- ◯ Not available

Resuscitation bag + mask size 2

- ◯ Available and functioning
- ◯ Available but not functioning
- ◯ Not available

Resucitation bag + mask size 3

◯ Available and functioning
◯ Available but not functioning
◯ Not available

Resucitation bag + mask size 4

◯ Available and functioning
◯ Available but not functioning
◯ Not available

Resucitation bag + mask size 5

◯ Available and functioning
◯ Available but not functioning
◯ Not available

CPAP

◯ Available and functioning
◯ Available but not functioning
◯ Not available

Hemoglobin testing

◯ Available and functioning
◯ Available but not functioning
◯ Not available

Blood type cross matching testing

◯ Available and functioning
◯ Available but not functioning
◯ Not available

Infusion set

◯ Available and functioning
◯ Available but not functioning
◯ Not available

Chest drain

◯ Available and functioning
◯ Available but not functioning
◯ Not available

Firm board/cardboard for splints

◯ Available and functioning
◯ Available but not functioning
◯ Not available

Oropharyngeal (guedel) airway

◯ Available and functioning
◯ Available but not functioning
◯ Not available

Suction pump

◯ Available and functioning
◯ Available but not functioning
◯ Not available

Micro nebulizer/spacer

◯ Available and functioning
◯ Available but not functioning
◯ Not available

Gloves

☐ Sterile
☐ Non-sterile
☐ Not available

Bandages

☐ Available and functioning
☐ Available but not functioning
☐ Not available

Nasogastric tubes

- ☐ Available different sizes
- ☐ Available only one size
- ☐ Not available

Emergency drugs

Dextrose 10%

- ◯ Yes
- ◯ No

Dextrose 25%

- ◯ Yes
- ◯ No

Dextrose 50%

- ◯ Yes
- ◯ No

Adrenaline

- ◯ Yes
- ◯ No

Diazepam

- ◯ Yes
- ◯ No

Paraldehyde

- ◯ Yes
- ◯ No

Phenobarbitone

- ◯ Yes
- ◯ No

Phenytoin

- ◯ Yes
- ◯ No

IV fluids (tick all that apply)

- ◯ NaCl
- ◯ Ringers Lactate
- ◯ Half-strength Darrow with 5% glucose
- ◯ None

IV benzyl PC

- ◯ Yes
- ◯ No

Gentamicin

- ◯ Yes
- ◯ No

Ceftriaxone

- ◯ Yes
- ◯ No

Ampicillin

- ◯ Yes
- ◯ No

Cloxacillin

- ◯ Yes
- ◯ No

IV artesunate

- ◯ Yes
- ◯ No

Dexametason/hydrocortisone

- ◯ Yes
- ◯ No

Salbutamol
- ☐ Oral
- ☐ For nebulisers
- ☐ None

Ipratropium
- ◯ Yes
- ◯ No

Aminofyllin
- ◯ Yes
- ◯ No

Magnesiumsulfate
- ◯ Yes
- ◯ No

Malnutrition F75/F100
- ◯ Yes
- ◯ No

ReSoMal
- ◯ Yes
- ◯ No

Facility ambulance
- ◯ Yes fuelled
- ◯ Yes without fuel
- ◯ Yes but broken
- ◯ No

Facility bicycle ambulance
- ◯ Yes
- ◯ No

Facility motorbike
- ◯ Yes fuelled
- ◯ Yes without fuel
- ◯ Yes but broken
- ◯ No

Facility fuel allowance
- ◯ Yes
- ◯ No

Facility airtime
- ◯ Yes
- ◯ No

How do you access ambulance services?

____
