# Supplementary material for: Capacity for delivery of paediatric emergency care and the current use of emergency triage, assessment and treatment in health facilities in the Busoga region, Uganda—A mixed methods study
Source: PLOS Glob Public Health. 2024 Sep 4;4(9):e0003666. doi: 10.1371/journal.pgph.0003666 (PMC11373804; doi:10.1371/journal.pgph.0003666)
Supplement: S2 File — (PDF) [file pgph.0003666.s003.pdf]

Health worker questionnaire

Record ID ______________________

Basic information

Has respondent provided his/her informed consent to participate in the study?
- ◯ Yes
- ◯ No

Today's date ______________________

What is your profession?
- ◯ Paediatrician
- ◯ Other specialist
- ◯ Medical doctor without specialisation
- ◯ Medical officer
- ◯ Clinical Officer
- ◯ Nurse
- ◯ Nurse assistant
- ◯ Midwife
- ◯ Attendant
- ◯ Other

For how long have you worked in this hospital? ______________________

Do you regularly manage severely sick children?
- ◯ Yes
- ◯ No

Emergency care training and knowledge

Have you ever done any training in management of acutely ill children, chose all that applies:
- ☐ No
- ☐ Yes ETAT
- ☐ Yes APLS
- ☐ Yes PALS
- ☐ Yes, Other

When did you last attend any training in the management of acutely sick children (estimate date) ______________________

Do you ever receive refresher training in management of acutely ill children at your workplace?
- ◯ Yes
- ◯ No

When was the last time your work place had a refresher training (estimate date) ______________________

Who provides the refresher training?
- ☐ Government
- ☐ District Health Office
- ☐ Research projects
- ☐ NGO projects
- ☐ Self initiated by staff at workplace
- ☐ Other

Have you ever been doing emergency management training with simulated patients?
- ◯ Yes
- ◯ No

Can you define ABC3D? Please write

____

How is hypoxemia defined?

____

How is hypoglycaemia defined?

____

What is the treatment for hypoglycaemia?

____

When should assessment for emergency signs take place?

- ☐ During routine examination
- ☐ When the child arrives at the facility
- ☐ When classifying the child's condition after examination
- ☐ After all children have arrived for the clinic

What three of these are signs of severe respiratory distress?

- ☐ Labored breathing
- ☐ Cough
- ☐ Head Nodding
- ☐ Very fast breathing
- ☐ Convulsions
- ☐ Fever

What is normal capillary refill time?

____

What is the treatment of convulsions?

An 8-month old weighs 6kg and is severely dehydrated - how much fluid should be given in the first hour (in mls)?

____

What does AVPU mean?

____

A child who is unconscious, with no trauma, but maintaining the airway should be put in which position?

____

What type of fluid can you give to initially treat shock?

____

At what flow (volume/time) should oxygen be started?

____

Below what age is a child always a priority (in months)?

____

Current practise

How many days per week do you see children clinically?

- ◯ Everyday
- ◯ 4-6 days/week
- ◯ 2-3 days/week
- ◯ 1 day/week
- ◯ Less than once a week

How many children do you see per week (estimate)?

____

How many children per week are classified as "severe illness"?

____

How many children per week do you refer?

____

Do you feel well prepared to manage critically ill children?

- ◯ I feel very well prepared
- ◯ Yes I feel well prepared
- ◯ No I do not feel well prepared
- ◯ No I do not at all feel well prepared

Do you get regular appreciation for your work?

- ☐ No
- ☐ Yes from patients
- ☐ Yes from colleagues
- ☐ Yes from superiors
- ☐ Yes from community

Do you have someone to consult in case you do not know how to manage a patient?

- ☐ No never
- ☐ Yes sometimes
- ☐ Yes most of the times
- ☐ Yes always

Who do you consult?

- ☐ Fellow colleagues
- ☐ Senior at same workplace
- ☐ Person at other hospital
- ☐ Other

Do you have any routines in place at your workplace for making sure that emergency equipment (trolley) is in place?

- ◯ Yes
- ◯ No

Is there any specific position at your workplace who is responsible for the emergency management routines (except from hospital management/head of department)?

- ◯ Yes
- ◯ No

When was the last time you managed a critically ill child?

- ◯ Today
- ◯ Yesterday
- ◯ This week
- ◯ This month
- ◯ More than a month ago

When you last managed a critically ill child, how many people assisted in the management of the patient?

- ◯ Only you
- ◯ 2-3 people
- ◯ 4-5 people
- ◯ >5 people

Were all the needed equipment/drugs available to handle the situation?

- ◯ Yes
- ◯ No

Was the emergency trolley/cupboard complete at the time?

- ◯ Yes
- ◯ No

Did you have any debriefing after you managed the sick child?

- ◯ Yes
- ◯ No

When was the last time you referred a child?

- ◯ Today
- ◯ Yesterday
- ◯ This week
- ◯ Last week
- ◯ This month
- ◯ More than a month ago

Have you ever called an ambulance for a sick child?

- ◯ Yes
- ◯ No

How long time did it take for the ambulance to reach your facility after you called them?

- ◯ < 30 minutes
- ◯ 30-60 minutes
- ◯ >60- 180 minutes
- ◯ >180 minutes

Does your work place triage paediatric patients?

- ◯ Yes
- ◯ No

Who conducts the triage?

- ☐ All staff
- ☐ Clinician (MD/MO/CO)
- ☐ Nurse
- ☐ Clinic assistant

What criteria do you use to triage?

- ◯ ETAT
- ◯ IMCI
- ◯ Child general appearance
- ◯ Caregiver report of severity
- ◯ Other

Does your workplace have a mechanism in place of reporting challenges you experience to the hospital and/or district lead?

- ◯ Yes
- ◯ No

How often do you get to communicate/report back to hospital/district leads?

- ◯ Weekly
- ◯ Monthly
- ◯ 4-6 times/year
- ◯ 1-3 times/year
- ◯ Less than yearly
