# Supplementary material for: Capacity for delivery of paediatric emergency care and the current use of emergency triage, assessment and treatment in health facilities in the Busoga region, Uganda—A mixed methods study
Source: PLOS Glob Public Health. 2024 Sep 4;4(9):e0003666. doi: 10.1371/journal.pgph.0003666 (PMC11373804; doi:10.1371/journal.pgph.0003666)
Supplement: S3 File — (PDF) [file pgph.0003666.s004.pdf]

Healthcare worker focus group discussions:

---

1. Welcome participants

2. Informed consent

    - Ensure all are aware of the audio recording
    - Assign participant numbers and re-iterate confidentiality
    - Complete participant information sheet

3. Current emergency treatment

- Can you give a recent example of child you saw, that you would describe as an emergency?
    - Probe: do others recognise this example, ask for multiple different ones, you can give an example
- Can you describe what you did with this child?
    - Probe: would everyone do the same, what would they do different
- What would you have liked to do in this situation (probe for differently than what was done, reasons for not doing this)?
- How did you feel about your management of this case (probe: went well, could have done differently)?
- How often do you see children like this?
- Do you use any tools or algorithms (guidelines) when assessing emergency cases?
- When managing emergency cases, how do you work? Is it alone, or in a team? Who is part of the team (Probe for positions, routines for who to be present, how are they called upon)?
- After handling an emergency, are there any routines for debriefing at your workplace?
- Do you have any procedure of reporting challenges you experience in the management of paediatric emergencies to the hospital lead/district lead?
- Do you ever get to discuss with hospital/district leads how management procedures could be improved (probe for frequency, experience of fruitful/non-fruitful discussions)?
- Are there any procedures in place for getting feedback on patient satisfaction of care/feedback from the public?

5. Current referral procedure

- How often do you refer a child to the hospital? For example, this week how many children has everyone referred?
- Can you describe the process of referral?
    - Probe: documentation, explaining to the caregiver, organising transport
- Do all caregivers complete the referral?
- Are there any barriers you face in referring children?
- Are there any benefits to referring children?
- Are there any mechanisms in place for getting feedback after you have referred a child (from the receiving uni)?

6. ETAT awareness

- How do you decide which order to see patients in your clinic?
- Have you heard of ETAT? Can you describe ETAT? (probe for positive/negative views)
- Do you know ETAT plus? What is the difference between ETAT and ETAT plus?

7. ETAT preparedness

- Has ETAT or ETAT plus been implemented in your workplace? (probe if other emergency management training such as APLS/PALS and ask follow up quesitons for that instead if needed – probe for when? By whom? Has it been followed up?)
- Do you have the equipment you need to manage acutely sick children? Can you give examples?
- Do you have the staff you need?
- Are there any barriers in your workplace to implementing ETAT? And barriers to adequate management of severely ill children?
- Are there any benefits to implementing ETAT in your setting?
