# Supplementary material for: Capacity for delivery of paediatric emergency care and the current use of emergency triage, assessment and treatment in health facilities in the Busoga region, Uganda—A mixed methods study
Source: PLOS Glob Public Health. 2024 Sep 4;4(9):e0003666. doi: 10.1371/journal.pgph.0003666 (PMC11373804; doi:10.1371/journal.pgph.0003666)
Supplement: S4 File — (DOCX) [file pgph.0003666.s005.docx]

| Variables |  | Unadjusted Analysis | | | | |
| --- | --- | --- | --- | --- | --- | --- |
|  |  | coefficient | 95% CI ^a^ | p-value | SE ^b^ | Goodness of fit |
| Profession | Paediatrician | Ref |  |  | Ref | R-squared = 0.3538  Adj R-squared = 0.3121 Root MSE = 3.4527 |
|  | Medical Officer | -2.8 | -8.0-2.5 | 0.300 | -0.9 |  |
|  | Clinical Officer | -4.3 ( | -9.4-0.7 | 0.091 | -2.0 |  |
|  | Nurse | -6.3 | -11.2- -1.2 | 0.015 | -4.3 |  |
|  | Nurse Assistant | -14.5 | -20.8 -8.2 | <0.001 | -11.6 |  |
|  | Midwife | -8.1 | -13.1- -3.1 | 0.002 | 5.7 |  |
|  | Other Training | -3.4 | -12.9- 0.9 | 0.086 | -3.3 |  |
| Years duty | <1 year | Ref |  |  | Ref | R-squared = 0.0803  Adj R-squared = 0.0613  Root MSE = 4.0332 |
|  | 1-3 year | 0.4 | -2.2-3.0 | 0.738 | -0.3 |  |
|  | >3 years | 2.7 | 0.9-5.1 | 0.036 | 1.3 |  |
| Emergency Training | None | Ref |  |  | Ref | R-squared = 0.1324  Adj R-squared = 0.1141  Root MSE = 3.9514 |
|  | ETAT^c^/APLS^d^/ PALS^e^/Other^f^ | 1.2 | -0.9-3.3) | 0.280 | 0.7 |  |
|  | ETAT^c^/APLS^d^/ PALS^e^/Other^f^ + refresher training | 3.5 | 1.7-5.3 | <0.001 | 2.3 |  |

^a^95%CI – 95 % confidence interval.

^b^SE – Standard Error.

^c^ETAT – Emergency Triage Assessment and Treatment.

^d^APLS – Advanced Paediatric Life Support.

^e^PALS – Pediatric Advanced Life Support.

^f^Other - ?

| Variables |  | Adjusted Analysis | | | |
| --- | --- | --- | --- | --- | --- |
|  |  | coefficient | 95%CI^b^ | p-value | Goodness of fit |
| Profession | Paediatrician | Ref |  |  | R-squared = 0.4521  Adj R-squared = 0.3891  Root MSE = 3.2813 |
|  | Medical Officer | -0.9 | -5.9-4.2 | 0.738 |  |
|  | Clinical Officer | -2.0 | -7.0-3.0 | 0.432 |  |
|  | Nurse | -4.3 | -9.2-0.6 | 0.087 |  |
|  | Nurse Assistant | -11.6 | -17.8- -5.3 | <0.001 |  |
|  | Midwife | 5.7 | -10.1- -0.8 | 0.024 |  |
|  | Other Training | -3.3 | -10.0-3.4 | 0.333 |  |
| Years duty | <1 year | Ref |  |  |  |
|  | 1-3 year | -0.3 | -2.5-2.0 | 0.805 |  |
|  | >3 years | 1.3 | -1.0-3.5 | 0.252 |  |
| Emergency Training | None | Ref |  |  |  |
|  | ETAT^a^/APLS^c^/ PALS^d^/Other^e^ | 0.7 | -1.0-2.5 | 0.421 |  |
|  | ETAT^a^/APLS^c^/ PALS^d^/Other^e^ + refresher training | 2.3 | 0.7-4.0 | 0.007 |  |

^a^ETAT – Emergency Triage Assessment and Treatment.

^b^95%CI – 95 % confidence interval.

^c^APLS – Advanced Paediatric Life Support.

^d^PALS – Pediatric Advanced Life Support.

^e^Other - ?
